# Longitudinal associations between BMI change and the risks of colorectal cancer incidence, cancer-relate and all-cause mortality among 81,388 older adults: BMI change and the risks of colorectal cancer incidence and mortality

**DOI:** 10.1186/s12885-019-6299-4

**Published:** 2019-11-11

**Authors:** Ji-Bin Li, Sheng Luo, Martin C. S. Wong, Cai Li, Li-Fen Feng, Jian-Hong Peng, Jing-Hua Li, Xi Zhang

**Affiliations:** 1 Department of Clinical Research, Sun Yat-sen University Cancer Center; State Key Laboratory of Oncology in South China, Collaborative Innovation Center for Cancer Medicine, Guangzhou, 510060 China; 2 Department of Biostatistics and Bioinformatics, Duke University School of Medicine, Durham, North Carolina 27710 USA; 3 JC School of Public Health and Primary Care, Faculty of Medicine, The Chinese University of Hong Kong, Sha Tin, Hong Kong, China; 4 Department of Statistics, Government Affairs Service Center of Health Commission of Guangdong Province, Guangzhou, 510060 China; 5 Department of Colorectal Surgery, Sun Yat-sen University Cancer Center; State Key Laboratory of Oncology in South China, Collaborative Innovation Center for Cancer Medicine, Guangzhou, 510060 China; 6 School of Public Health, Sun Yat-sen University, Guangzhou, 510080 China; 7 Clinical Research Unit, Xin Hua Hospital, Shanghai Jiao Tong University School of Medicine, 1665 Kongjiang Road, Kejiao Building 233B, Shanghai, 200092 China

**Keywords:** BMI change, Colorectal cancer risk, Mortality, Older adults, Longitudinal association

## Abstract

**Background:**

It remains controversial whether weight change could influence the risks of colorectal cancer (CRC) and mortality. This study aimed to quantify the associations between full-spectrum changes in body mass index (BMI) and the risks of colorectal cancer (CRC) incidence, cancer-related and all-cause mortality among midlife to elder population.

**Methods:**

A total of 81,388 participants who were free of cancer and aged 55 to 74 years from the Prostate, Lung, Colorectal, and Ovarian (PLCO) screening program were involved. The percentage change of BMI was calculated as (BMI in 2006 - BMI at baseline)/BMI at baseline, and was categorized into nine groups: decrease (≥ 15.0%, 10.0–14.9%, 5.0–9.9%, 2.5–4.9%), stable (decrease/increase < 2.5%), increase (2.5–4.9%, 5.0–9.9%, 10.0–14.9%, ≥ 15.0%). The associations between percentage change in BMI from study enrolment to follow-up (median: 9.1 years) and the risks of CRC and mortality were evaluated using Cox proportional hazard regression models.

**Results:**

After 2006, there were 241 new CRC cases, 648 cancer-related deaths, and 2361 all-cause deaths identified. Overall, the associations between BMI change and CRC incidence and cancer-related mortality, respectively, were not statistically significant. Compared with participants whose BMI were stable, individuals who had a decrease in BMI were at increased risk of all-cause mortality, and the HRs were 1.21 (95% CI: 1.03–1.42), 1.65 (95% CI: 1.44–1.89), 1.84 (95% CI: 1.56–2.17), and 2.84 (95% CI: 2.42–3.35) for 2.5–4.9%, 5.0–9.9%, 10.0–14.9%, and ≥ 15.0% decrease in BMI, respectively. An L-shaped association between BMI change and all-cause mortality was observed. Every 5% decrease in BMI was associated with a 27% increase in the risk of all-cause mortality (HR = 1.27, 95% CI: 1.22–1.31, *p* < 0.001). The results from subgroups showed similar trends.

**Conclusions:**

A decrease in BMI more than 5% shows a significantly increased risk of all-cause mortality among older individuals; but no significant association between increase in BMI and all-cause mortality. These findings emphasize the importance of body weight management in older population, and more studies are warranted to evaluate the cause-and-effect relationship between changes in BMI and cancer incidence/mortality.

## Background

Overweight and obesity is the fifth leading cause of overall mortality, accounting for at least 2.8 million adult deaths each year [1]. As a major global health burden, excess adiposity is a well-established risk factor for various chronic diseases, including cardiovascular diseases, cancers (i.e., cancers of the breast, colorectal, endometrial, kidney, and prostate), and all-cause mortality [2–4]. Obesity is implicated in carcinogenesis, and may affect cancer development through alterations in metabolism of insulin, insulin-like growth factors, chronic inflammation, adipokines and steroid hormones [5, 6]. It was estimated that 3.9% of all cancers (544,300 cases) in 2012 were attributable to excess adiposity in 2002 [7].

Colorectal cancer (CRC), the third most commonly diagnosed cancer in men and the second in women, is an obesity-related cancer [8], with a worldwide estimate of 1.8 million cases in 2018 [9]. Epidemiological evidence has demonstrated that higher body mass index (BMI) in childhood or young adulthood increases the risk of CRC and mortality [8, 10, 11]. In addition to excess adiposity, weight change has been frequently examined in relation to CRC morbidity and mortality. However, the findings remains inconclusive. Four systemic review and meta-analyses summarized that adulthood weight gain, measured by body weight or BMI, was significantly associated with a higher risk of CRC, and the estimated increase in the risk of CRC varied from 3 to 9% by per 5-unit weight gain [12–15]. Karahalios A. et al ^15^ further revealed in a meta-analysis that weight gain from early adulthood to midlife but not from midlife to older age was associated with an increased risk of CRC. However, a recent study from the Melbourne Collaborative Cohort Study reported a non-significant association between a 5 kg increase in weight and the risk of incident CRC [16].

Similarly, investigations on weight loss are challenging, as studies of its impact on the risk of cancer and mortality are sparse and provided mixed conclusions [17]. A study among Japanese population found that the incident rates of colorectal adenoma in subjects with weight reduction (more than 7% weight loss) was significantly lower than that in those having no weight loss [18]. With respect to mortality, a recent meta-analysis of prospective studies reported that both weight gain and weight loss were associated with an increased risk of all-cause mortality in the middle-aged populations and in older adults [19]. However, the relation between weight gain or weight loss and the risk of mortality was not statistically significant.

Further, there is a definite knowledge gap for public health policies and cancer prevention strategies in the associations between full spectrum of weight change, including increase and decrease of weight, and the risks of CRC incidence, cancer-related and all-cause mortality among the midlife to elderly population, given that weight change from midlife to older age might involve different mechanisms (e.g., due to decrease in muscle mass and increase in fat mass), as compared to early adulthood to midlife [19, 20]. It is still unclear whether a weight change across the midlife to elderly period relates to the subsequent short-term risk of CRC incidence, cancer-related and all-cause mortality. Therefore, in this study, we analyzed the data from Prostate, Lung, Colorectal, and Ovarian (PLCO) screening program to systematically examine the associations between full spectrum of BMI change from 1993 to 2006 and the subsequent short-term risk of CRC incidence, cancer-related and all-cause mortality.

## Methods

### Study design and population

The PLCO cancer screening program is a randomized controlled, multicenter trial, which enrolled 154,897 participants aged 55 to 74 years from 1993 to 2001 in ten centers across the United States. All centers ended the recruitment at the end of 2001.

The PLCO study was designed as previously described [21, 22]. In brief, eligible participants were randomly assigned to either a usual care arm or screening arm. Participants in the screening arm were offered flexible sigmoidoscopy at baseline and at 3 years (for those who underwent randomization before April 1995) or at 5 years, and participants in the control arm only received routine health care from their health care providers. All participants completed baseline questionnaires to collect their demographics variables, smoking status, family history of any cancer in their first-degree relatives, personal history of chronic diseases (including hypertension, heart attack, stroke, emphysema, diabetes, arthritis, and osteoporosis), as well as body weight and height. A follow-up survey was conducted to update baseline information and anthropometric measures in 2006. All participants were followed for incident cancer and cause-specific deaths. The PLCO study protocol was approved by the Institutional Review Board of the National Cancer Institute and the participating centers. All participants provided written consent upon enrollment.

**Fig. 1:**
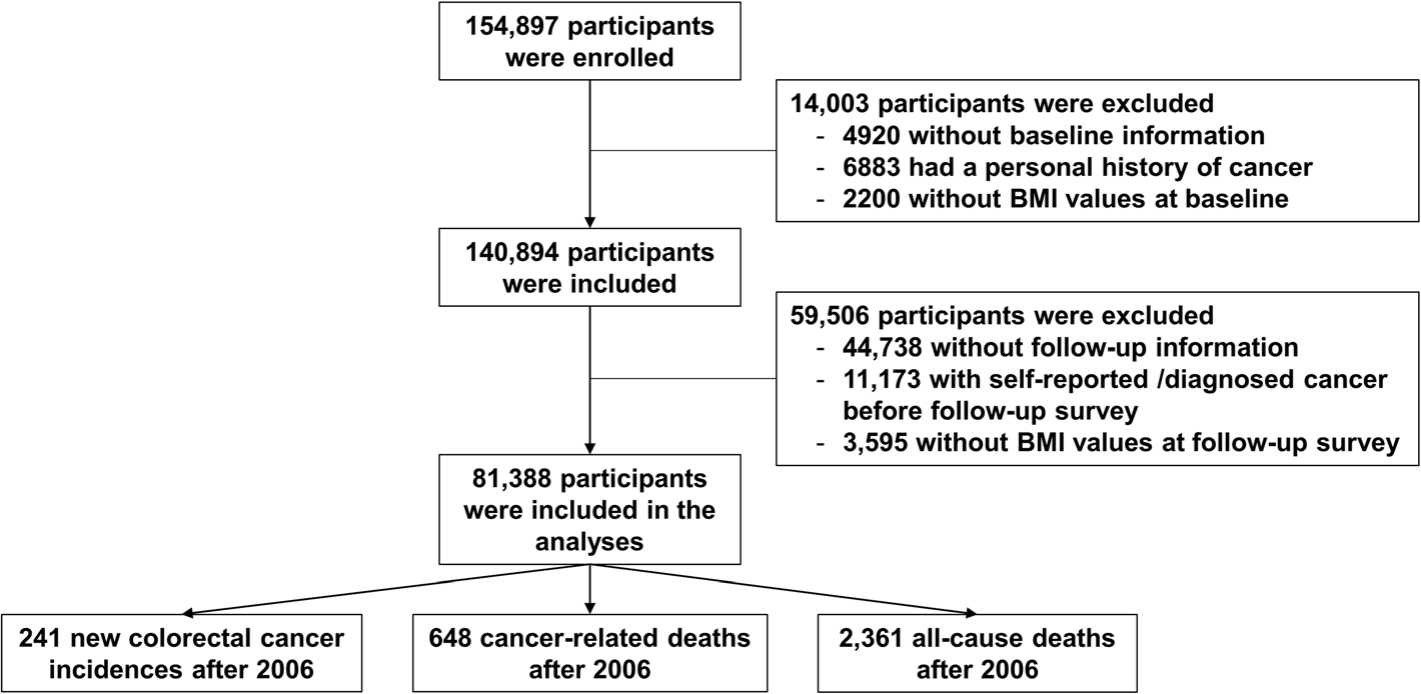
Flowchart of the participants’ selection

Eligible participants included subjects who provided a valid baseline and follow-up questionnaire with no missing values on their height or weight; those who had no history of cancer; and those who had no diagnosis of cancer before 2006. The selection process is illustrated in Fig. 1, and a total of 81,388 from 154,897 (52.54%) participants were eligible.

### BMI assessment

Height (in feet and inches) and body weight (in pounds) were self-reported at the study entry interview, and body weight was updated in 2006. Body mass index (BMI) was calculated as the weight (kg) divided by the squared of the height (m). The BMI was categorized into four groups based on World Health Organization guideline: underweight (less than 18.5 kg/m^2^), normal weight (18.5 to 24.9 kg/m^2^), overweight (25.0 to 29.9 kg/m^2^), and obesity (30 kg/m^2^ or greater). The percent change (%) in BMI was calculated as

$$ \frac{BMI\  at\ 2006- BMI\  at\  study\ entry}{BMI\  at\  study\ entry}\times 100\% $$

The percent change (%) in BMI was categorized into nine categories: decrease (≥15.0%, 10.0–14.9%, 5.0–9.9%, 2.5–4.9%), stable (decrease/increase < 2.5%), increase (2.5–4.9%, 5.0–9.9%, 10.0–14.9%, ≥15.0%). Stable category was used as a reference group in data analyses.

### Outcome ascertainment

Incident CRC was ascertained by proper diagnostic evaluation [22]. Cause-specific mortality was collected by active follow-up using annual study update questionnaires, linkage to the National Death Index, medical records and/or death certificate, whilst death review process was conducted in order to provide accurate assessment of these mortality events [23, 24].

### Statistical analyses

Continuous variables were described as means ± standard deviations (SD), or the medians (interquartile ranges) where appropriate, and categorical variables were presented as proportions. For CRC incidence, follow-up time (in years) was measured from the date of trial entry (randomization) to the date of CRC diagnosis, death, or last follow-up (censoring date), and for mortality, the follow-up time period (in years) were calculated as the time interval from the date of trial entry (randomization) to the date of any-cause mortality or the last date of follow-up (censoring date), whichever came first. Data were censored on December 31, 2009, or at 13th years of randomization, whichever occurred first [25].

We estimated the percent change of BMI in relation to the risk of CRC incidence, cancer-related mortality, and all-cause mortality among all participants and subgroups, including sex, age at study entry (< 65 years old and ≥ 65 years old), BMI status at study entry (< 25 kg/m^2^, 25–29.9 kg/m^2^, and ≥ 30 kg/m^2^), year of study enrolment (1993–1997 and 1998–2001), and years from study entry to 2006 (≤ 10 years and > 10 years). The interaction among variables, including change in BMI, sex, age at study entry, BMI status at study entry, year of study enrolment, and years from study entry to 2006, were tested by adding the product terms in statistical models. The associations between change in BMI status from study entry to 2006 and the risks of CRC incidence, cancer-related mortality and all-cause mortality were also examined. Hazard ratios (HRs) and 95% confidence intervals (CIs) were calculated by Cox proportional hazards regression models after adjustment of potential confounders, with proportional hazards assumption confirmed based on the Schoenfeld residuals [26].

Tests for linear trend were performed using percent change in BMI as a continuous variable in the models; tests for linear trend across decrease in BMI were restricted to participants who had a decreased BMI, and tests for trend across increase in BMI were restricted to participants who had an increased BMI from study entry to 2006. Possible nonlinear relationships of percentage change in BMI to the risk of CRC incidence, cancer-related mortality, and all-cause mortality were tested non-parametrically with restricted cubic spline regression models with three knots at 25th, 50th, and 75th percentiles. The non-linearity among variables was tested using the likelihood ratio test, comparing the model with the linear term only versus the model with the linear and cubic spline terms.

All models were adjusted for sex, age at randomization, ethnicity/race, education level, family annual income, marital status, physical activity level, smoking status, history of any cancer in their first-degree relatives, screening arm, personal history of chronic diseases (i.e., hypertension, heart attack, stroke, emphysema, diabetes, arthritis, and osteoporosis), and BMI value at study entry (continuous).

All analyses were performed using the SAS software (version 9.4, SAS Institute Inc., Cary, NC). All *p* values were based on two-sided tests and were considered statistically significant at *p* ≤ 0.05.

## Results

### Participants’ characteristics and BMI change

Among 81,388 participants, there were 241 new CRC cases, 648 cancer-related deaths, and 2361 all-cause deaths observed from 2006 to 2009. The mean age was 62 years (SD: 5) at study entry. The median follow-up time was 12.5 years (range: 5.3 to 13.0). Participants’ characteristics across categories of percentage change in BMI were shown in Table 1. The mean percent change in BMI was 1.02% (men: 0.96%; women: 1.07%) from study entry to 2006. Around a third (32.1%) of the participants had a decrease in BMI greater than 2.5%. The ratio of men to women was 0.9:1, and majority of the participants (91.2%) were non-Hispanic white. The top three types of chronic diseases reported by the participants were hypertension (49.29%), arthritis (46.56%), and osteoporosis (15.03%). Around 23.0% of the participants were obese, and 42.8% were overweight at study entry. Participants who had a decrease in BMI were more likely to be women, older, obese at study entry, and more active than 10 years ago; while those with an increase in BMI were more likely to have reported normal BMI at study entry.

**Table 1:** Participants’ characteristics stratified by categories of percentage change in BMI

	Total	BMI decrease				Stable BMI (+/− 2.5%)	BMI increase			
		≥15%	10–14.9%	5–9.9%	2.5–4.9%		2.5–4.9%	5–9.9%	10–14.9%	≥15%
All	81,388	2783 (3.42)	4128 (5.07)	10,122 (12.44)	9098 (11.18)	22,187 (27.26)	10,511 (12.91)	12,978 (15.95)	5471 (6.72)	4110 (5.05)
Percentage change of BMI, Mean ± SD	1.02 ± 9.58	−21.19 ± 6.36	−12.12 ± 1.41	−7.14 ± 1.41	−3.62 ± 0.71	0.04 ± 1.36	3.57 ± 0.75	7.14 ± 1.44	12.11 ± 1.40	24.23 ± 14.05
Sex										
Men	38,250 (47.00)	963 (34.60)	1593 (38.59)	4823 (47.65)	4525 (49.74)	11,363 (51.21)	5144 (48.94)	5999 (46.22)	2313 (42.28)	1527 (37.15)
Women	43,138 (53.00)	1820 (65.40)	2535 (61.41)	5299 (52.35)	4573 (50.26)	10,824 (48.79)	5367 (51.06)	6979 (53.78)	3158 (57.72)	2583 (62.85)
Age at study entry (Year), Mean ± SD	61.92 ± 5.14	63.18 ± 5.51	63.18 ± 5.42	62.68 ± 5.30	62.38 ± 5.23	61.81 ± 5.10	61.54 ± 5.05	61.36 ± 4.93	61.11 ± 4.86	61.05 ± 4.83
≤ 59	30,558 (37.55)	836 (30.04)	1237 (29.97)	3292 (32.52)	3087 (33.93)	8479 (38.22)	4120 (39.20)	5351 (41.23)	2361 (43.15)	1795 (43.67)
60–64	25,825 (31.73)	843 (30.29)	1222 (29.60)	3082 (30.45)	2910 (31.99)	7043 (31.74)	3384 (32.19)	4252 (32.76)	1775 (32.44)	1314 (31.97)
65–69	16,778 (20.61)	622 (22.35)	1031 (24.98)	2425 (23.96)	2055 (22.59)	4539 (20.46)	2050 (19.50)	2377 (18.32)	947 (17.31)	732 (17.81)
≥ 70	8227 (10.11)	482 (17.32)	638 (15.46)	1323 (13.07)	1046 (11.50)	2126 (9.58)	957 (9.10)	998 (7.69)	388 (7.09)	269 (6.55)
Ethnic group										
White (non-Hispanic)	74,201 (91.17)	2513 (90.30)	3701 (89.66)	9127 (90.17)	8244 (90.61)	20,172 (90.92)	9617 (91.49)	11,938 (91.99)	5075 (92.76)	3814 (92.80)
Black (non-Hispanic)	2511 (3.09)	131 (4.71)	189 (4.58)	353 (3.49)	280 (3.08)	646 (2.91)	271 (2.58)	359 (2.77)	166 (3.03)	116 (2.82)
Others (i.e., Asian, pacific islander, etc.)	4676 (5.75)	139 (4.99)	238 (5.77)	642 (6.34)	574 (6.31)	1369 (6.17)	623 (5.93)	681 (5.25)	230 (4.20)	180 (4.38)
Educational level										
High school graduate or less	22,383 (27.50)	898 (32.27)	1262 (30.57)	2857 (28.23)	2477 (27.23)	5803 (26.15)	2632 (25.04)	3600 (27.74)	1593 (29.12)	1261 (30.68)
Some college	27,943 (34.33)	987 (35.47)	1448 (35.08)	3540 (34.97)	3149 (34.61)	7365 (33.20)	3604 (34.29)	4453 (34.31)	1897 (34.67)	1500 (36.50)
College graduate	14,696 (18.06)	429 (15.42)	659 (15.96)	1797 (17.75)	1613 (17.73)	4260 (19.20)	2019 (19.21)	2355 (18.15)	944 (17.25)	620 (15.09)
Postgraduate	16,229 (19.94)	462 (16.60)	751 (18.19)	1916 (18.93)	1842 (20.25)	4713 (21.24)	2241 (21.32)	2556 (19.69)	1026 (18.75)	722 (17.57)
Unknown	137 (0.17)	7 (0.25)	8 (0.19)	12 (0.12)	17 (0.19)	46 (0.21)	15 (0.14)	14 (0.11)	11 (0.20)	7 (0.17)
Family annual income										
< $20, 000	8819 (10.84)	519 (18.65)	643 (15.58)	1198 (11.84)	934 (10.27)	1987 (8.96)	964 (9.17)	1288 (9.92)	673 (12.30)	613 (14.91)
$20, 000–49, 000	29,878 (36.71)	1018 (36.58)	1571 (38.06)	3758 (37.13)	3399 (37.36)	7844 (35.35)	3757 (35.74)	4872 (37.54)	2071 (37.85)	1588 (38.64)
$50, 000–99, 000	21,274 (26.14)	561 (20.16)	901 (21.83)	2459 (24.29)	2433 (26.74)	6142 (27.68)	3001 (28.55)	3461 (26.67)	1353 (24.73)	963 (23.43)
≥ $100, 000	7028 (8.64)	127 (4.56)	243 (5.89)	783 (7.74)	774 (8.51)	2249 (10.14)	1034 (9.84)	1120 (8.63)	451 (8.24)	247 (6.01)
Not answered	14,389 (17.68)	558 (20.05)	770 (18.65)	1924 (19.01)	1558 (17.12)	3965 (17.87)	1755 (16.70)	2237 (17.24)	923 (16.87)	699 (17.01)
Marital status										
Married/cohabiting	63,693 (78.26)	2070 (74.38)	3200 (77.52)	8006 (79.10)	7288 (80.11)	17,833 (80.38)	8308 (79.04)	10,097 (77.80)	4017 (73.42)	2874 (69.93)
Single	17,586 (21.61)	709 (25.48)	922 (22.34)	2104 (20.79)	1799 (19.77)	4319 (19.47)	2193 (20.86)	2867 (22.09)	1442 (26.36)	1231 (29.95)
Missing	109 (0.13)	4 (0.14)	6 (0.15)	12 (0.12)	11 (0.12)	35 (0.16)	10 (0.10)	14 (0.11)	12 (0.22)	5 (0.12)
Physical active level compared with 10 years ago										
About the same	32,228 (39.60)	845 (30.36)	1443 (34.96)	4173 (41.23)	3981 (43.76)	9862 (44.45)	4471 (42.54)	4705 (36.25)	1721 (31.46)	1027 (24.99)
Less active	41,245 (50.68)	1482 (53.25)	2082 (50.44)	4655 (45.99)	4140 (45.50)	10,218 (46.05)	5212 (49.59)	7237 (55.76)	3403 (62.20)	2816 (68.52)
More active	6788 (8.34)	399 (14.34)	530 (12.84)	1122 (11.08)	843 (9.27)	1831 (8.25)	692 (6.58)	880 (6.78)	282 (5.15)	209 (5.09)
Not answered	1127 (1.38)	57 (2.05)	73 (1.77)	172 (1.70)	134 (1.47)	276 (1.24)	136 (1.29)	156 (1.20)	65 (1.19)	58 (1.41)
Family history of cancer in their first relatives										
No	35,795 (43.98)	1195 (42.94)	1777 (43.05)	4457 (44.03)	3997 (43.93)	9853 (44.41)	4580 (43.57)	5797 (44.67)	2363 (43.19)	1776 (43.21)
Yes	45,386 (55.76)	1582 (56.85)	2342 (56.73)	5644 (55.76)	5070 (55.73)	12,280 (55.35)	5900 (56.13)	7149 (55.09)	3096 (56.95)	2323 (56.52)
Missing	207 (0.25)	6 (0.22)	9 (0.22)	21 (0.21)	31 (0.34)	54 (0.24)	31 (0.29)	32 (0.25)	12 (0.22)	11 (0.27)
Smoking status at study entry										
Never smoker	39,837 (48.95)	1336 (48.01)	1999 (48.43)	4988 (49.28)	4553 (50.04)	11,148 (50.25)	5321 (50.62)	6287 (48.44)	2457 (44.91)	1748 (42.53)
Current smoker	34,725 (42.67)	1172 (42.11)	1746 (42.30)	4302 (42.50)	3917 (43.05)	9485 (42.75)	4433 (42.17)	5579 (42.99)	2386 (43.61)	1705 (41.48)
Former smoker	6816 (8.37)	274 (9.85)	383 (9.28)	829 (8.19)	626 (6.88)	1554 (7.00)	756 (7.19)	1110 (8.55)	628 (11.48)	656 (15.96)
Missing	10 (0.01)	1 (0.04)	0	3 (0.03)	2 (0.02)	0	1 (0.01)	2 (0.02)	0	1 (0.02)
BMI at study entry (kg/m^2^), Mean ± SD	27.21 ± 4.78	31.31 ± 6.66	29.21 ± 5.39	28.07 ± 4.92	27.22 ± 4.57	26.79 ± 4.43	26.49 ± 4.33	26.76 ± 4.37	26.75 ± 4.57	26.49 ± 5.04
< 18.5 kg/m^2^	515 (0.63)	–	12 (0.29)	52 (0.51)	31 (0.34)	111 (0.50)	56 (0.53)	63 (0.49)	33 (0.60)	157 (3.82)
18.5–24.9 kg/m^2^	27,317 (33.56)	402 (14.44)	861 (20.86)	2688 (26.56)	2901 (31.89)	8055 (36.31)	4123 (39.23)	4668 (35.97)	2067 (37.78)	1552 (37.76)
25–29.9 kg/m^2^	34,822 (42.79)	955 (34.32)	1684 (40.79)	4443 (43.89)	4136 (45.46)	9626 (43.39)	4442 (42.26)	5773 (44.48)	2272 (41.53)	1491 (36.28)
≥ 30 kg/m^2^	18,734 (23.02)	1426 (51.24)	1571 (38.06)	2939 (29.04)	2030 (22.31)	4395 (19.18)	1890 (17.98)	2474 (19.06)	1099 (20.09)	910 (22.14)
BMI at follow-up (kg/m^2^), Mean ± SD	27.40 ± 5.03	24.57 ± 5.06	25.66 ± 4.74	26.06 ± 4.56	26.23 ± 4.39	26.80 ± 4.44	27.43 ± 4.50	28.67 ± 4.72	29.99 ± 5.14	32.77 ± 6.58
< 18.5 kg/m^2^	759 (0.93)	230 (8.26)	116 (2.81)	170 (1.68)	87 (0.96)	113 (0.51)	23 (0.22)	17 (0.13)	2 (0.04)	1 (0.02)
18.5–24.9 kg/m^2^	26,555 (32.63)	1430 (51.38)	1939 (46.97)	4411 (43.58)	3766 (41.39)	8117 (36.58)	3192 (30.37)	2607 (20.09)	778 (14.22)	315 (7.66)
25–29.9 kg/m^2^	33,972 (41.74)	737 (26.48)	1404 (33.94)	3855 (39.09)	3778 (41.53)	9564 (43.11)	4853 (46.17)	6262 (48.25)	2290 (41.86)	1232 (29.98)
≥ 30 kg/m^2^	20,102 (24.70)	386 (13.87)	672 (16.28)	1686 (16.66)	1467 (16.12)	4393 (19.80)	2443 (23.24)	4092 (31.53)	2401 (43.89)	2562 (62.34)
Arm										
Screening	41,703 (51.24)	1444 (51.89)	2134 (51.70)	5220 (51.57)	4698 (51.64)	11,457 (51.64)	5374 (51.13)	6616 (50.98)	2711 (49.55)	2049 (49.85)
Usual care	39,685 (78.76)	1339 (48.11)	1994 (48.30)	4902 (48.43)	4400 (48.36)	10,730 (48.36)	5137 (48.87)	6362 (49.02)	2760 (50.45)	2061 (50.15)
Chronic diseases at study entry/follow-up										
Hypertension	40,115 (49.29)	1571 (56.45)	2153 (52.16)	5013 (49.53)	4282 (47.07)	10,304 (46.44)	4981 (47.39)	6509 (50.15)	2929 (53.54)	2373 (57.74)
Heart attack	6919 (8.50)	331 (11.89)	437 (10.59)	938 (9.27)	728 (8.00)	1655 (7.46)	796 (7.57)	1067 (8.22)	503 (9.19)	464 (11.29)
Stroke	4177 (5.13)	250 (8.98)	318 (7.70)	625 (6.17)	461 (5.07)	917 (4.13)	456 (4.34)	595 (4.58)	293 (5.36)	262 (6.37)
Emphysema	2742 (3.37)	145 (5.21)	188 (4.55)	357 (3.53)	263 (2.89)	573 (2.58)	289 (2.75)	404 (3.11)	233 (4.26)	290 (7.06)
Diabetes	10,463 (12.86)	683 (24.54)	865 (20.95)	1755 (17.34)	1164 (12.79)	2309 (10.41)	964 (9.17)	1346 (10.37)	663 (12.12)	714 (17.37)
Arthritis	37,895 (46.56)	1525 (54.80)	2117 (51.28)	4785 (47.27)	4086 (44.91)	9864 (44.46)	4643 (44.17)	5977 (46.05)	2717 (49.66)	2181 (53.07)
Osteoporosis	12,233 (15.03)	559 (20.09)	700 (16.96)	1470 (14.52)	1288 (14.16)	3111 (14.02)	1513 (14.39)	1908 (14.70)	944 (17.25)	740 (18.00)

Data were presented as frequency (percentage) unless specified. BMI: body mass index; SD: standard deviation

### BMI change in relation to the risk of incident CRC

**Fig. 2:**
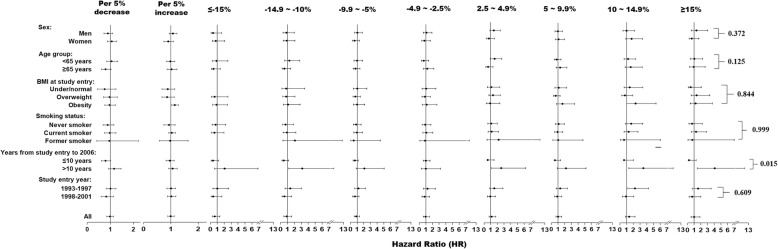
Associations between percentage change in BMI from study enrolment (1993–2001) to follow-up (2006) and the risk of CRC. The reference value (HR = 1) was set at percentage change between − 2.5 and 2.5%. HRs were estimated by cox proportional hazard model adjusted of sex, age, race, education level, family annual income, marital status, physical activity level, family history of cancer in their first-degree relatives, smoking status, screening arm, history of chronic diseases (i.e., hypertension, heart attack, stroke, emphysema, diabetes, arthritis, and osteoporosis), and BMI value at study entry (continuous)

Overall, the association between percentage change in BMI and the risk of CRC was not statistically significant. The results of subgroup analyses showed that a 5% increase in BMI was associated with 14% increase in the risk of CRC (HR = 1.14, 95% CI: 1.03–1.27; *p* = 0.015) among participants who were obese at study entry. There was significant interaction between BMI change and years from study entry to 2006. Among those who were enrolled in the cohort for more than 10 years, as compared to those with stable BMI, there were an increased risk of CRC for those with a 10–14.9% decrease in BMI (HR = 3.12–95%CI: 1.18, 8.24; *p* = 0.021), and those with 2.5–4.9% (HR = 2.57, 95% CI: 1.07–6.22; *p* = 0.036), 10–14.9% (HR = 3.49, 95% CI: 1.34–9.11; *p* = 0.011), and ≥ 15% (HR = 4.06, 95%CI: 1.48–11.13; *p* = 0.006) increase in BMI. The associations between BMI change and the risk of CRC incidence were not statistically significant in other subgroups (Fig. 2). Similarly, the associations between changes in BMI status and the risk of CRC incidence were not statistically significant (Table 2).

**Table 2:** Associations between change in BMI status and the risk of CRC incidence, cancer-related mortality, and all-cause mortality among all participants stratified by BMI status at study entry

BMI change	No. of participants	Incident CRC				Cancer-related mortality				All-cause mortality			
		No. of cases	HR	95% CI	*p*	No. of cases	HR	95% CI	*p*	No. of cases	HR	95% CI	*p*
Under/normal weight at study entry													
Under/normal weight at follow-up	21,749	47	1.00	–	–	163	1.00	–		574	1.00	–	–
Overweight at follow-up	5781	19	1.24	0.69, 2.23	0.46	54	1.07	0.77, 1.50	0.67	113	**0.69**	**0.56, 0.85**	**< 0.001**
Obesity at follow-up	302	0	–	–	–	5	1.52	0.61, 3.77	0.37	12	1.13	0.63, 2.01	0.69
Overweight at study entry													
Under/normal weight at follow-up	5244	16	1.02	0.58, 1.81	0.943	41	1.06	0.74, 1.51	0.76	261	**1.85**	**1.59, 2.16**	**< 0.001**
Overweight at follow-up	24,533	68	1.00	–	–	176	1.00	–	–	603	1.00	–	–
Obesity at follow-up	5044	13	0.93	0.49, 1.74	0.814	36	0.82	0.56, 1.20	0.315	132	0.86	0.70, 1.04	0.12
Obesity at study entry													
Under/normal weight at follow-up	321	2	1.68	0.41, 7.00	0.472	4	1.35	0.50, 3.70	0.555	27	**2.59**	**1.75, 3.85**	**< 0.001**
Overweight at follow-up	3658	13	0.93	0.50, 1.74	0.812	38	1.20	0.82, 1.76	0.361	147	**1.37**	**1.13, 1.67**	**0.002**
Obesity at follow-up	14,755	63	1.00	–	–	131	1.00	–	–	492	1.00	–	–

*CRC*: Colorectal cancer; *BMI*: Body mass index; *HR*: Hazard ratio; 95% CI: 95% confidence interval. HRs were adjusted by cox regression models for sex, age, race, education level, family annual income, marital status, physical activity level, family history of cancer, smoking status, screening arm, history of chronic diseases (i.e., hypertension, heart attack, stroke, emphysema, diabetes, arthritis, and osteoporosis), and baseline BMI value (continuous) Boldface means statistically significance

**Fig. 3:**
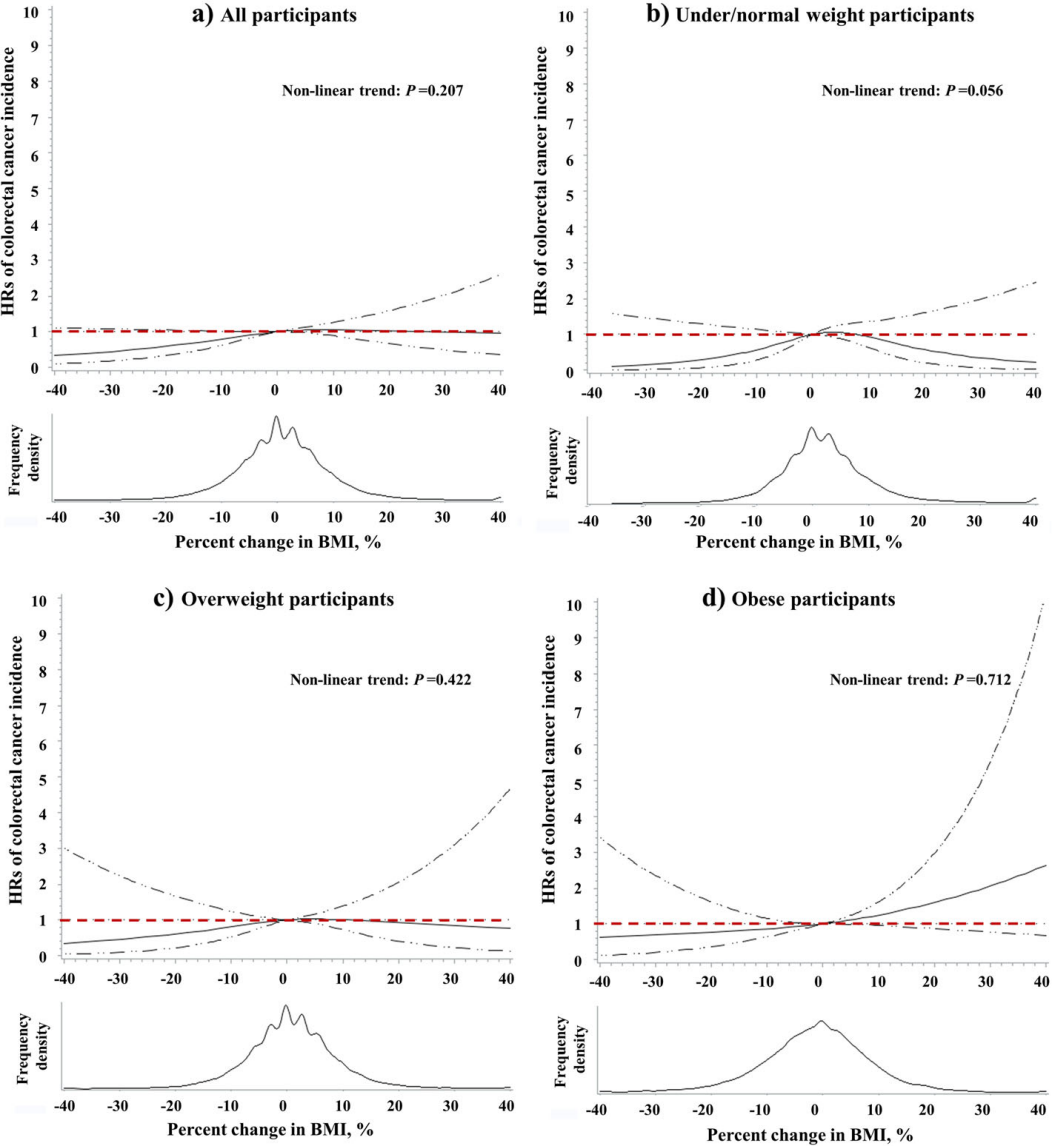
Restricted spline curves for the associations between percentage change in BMI and the risk of CRC among overall (**a**), under/normal weight (**b**), overweight (**c**) and obese (**d**) participants. The solid curve represents multivariate-adjusted HRs calculated by restricted cubic splines with 3 knots at the 25th, 50th, and 75th of the percentage change in BMI; the solid dashed lines represent 95% confidence interval. The reference value (HR = 1) was set at percentage change in BMI = 0. HRs were estimated by cox proportional hazard model adjusted of sex, age, race, education level, family annual income, marital status, physical activity level, family history of cancer in their first-degree relatives, smoking status, screening arm, history of chronic diseases (i.e., hypertension, heart attack, stroke, emphysema, diabetes, arthritis, and osteoporosis), and BMI value at study entry (continuous)

The nonlinear relationship between BMI change and the risk of CRC were not statistically significant among overall (*p* for nonlinear trend = 0.207; Fig. 3a); among those who were under/normal weight (*p* for nonlinear trend = 0.056; Fig. 3b), overweight (*p* for nonlinear trend = 0.422; Fig. 3c), and obese (*p* for nonlinear trend = 0.712; Fig. 3d) participants, after adjustment of covariates.

### BMI change in relation to cancer-related mortality

**Fig. 4:**
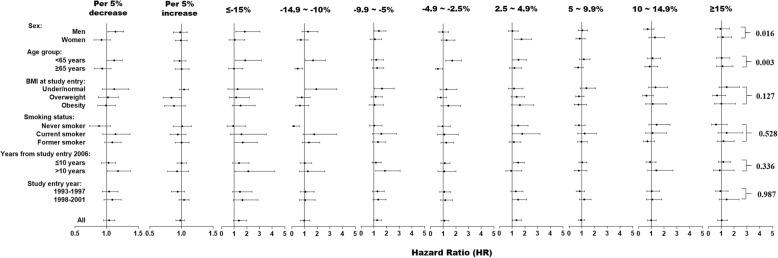
Associations between percentage change in BMI from study enrolment (1993–2001) to follow-up (2006) and the risk of cancer-related mortality. The reference value (HR = 1) was set at percentage change between − 2.5 and 2.5%. HRs were estimated by cox proportional hazard model adjusted of sex, age, race, education level, family annual income, marital status, physical activity level, family history of cancer in their first-degree relatives, smoking status, screening arm, history of chronic diseases (i.e., hypertension, heart attack, stroke, emphysema, diabetes, arthritis, and osteoporosis), and BMI value at study entry (continuous)

**Fig. 5:**
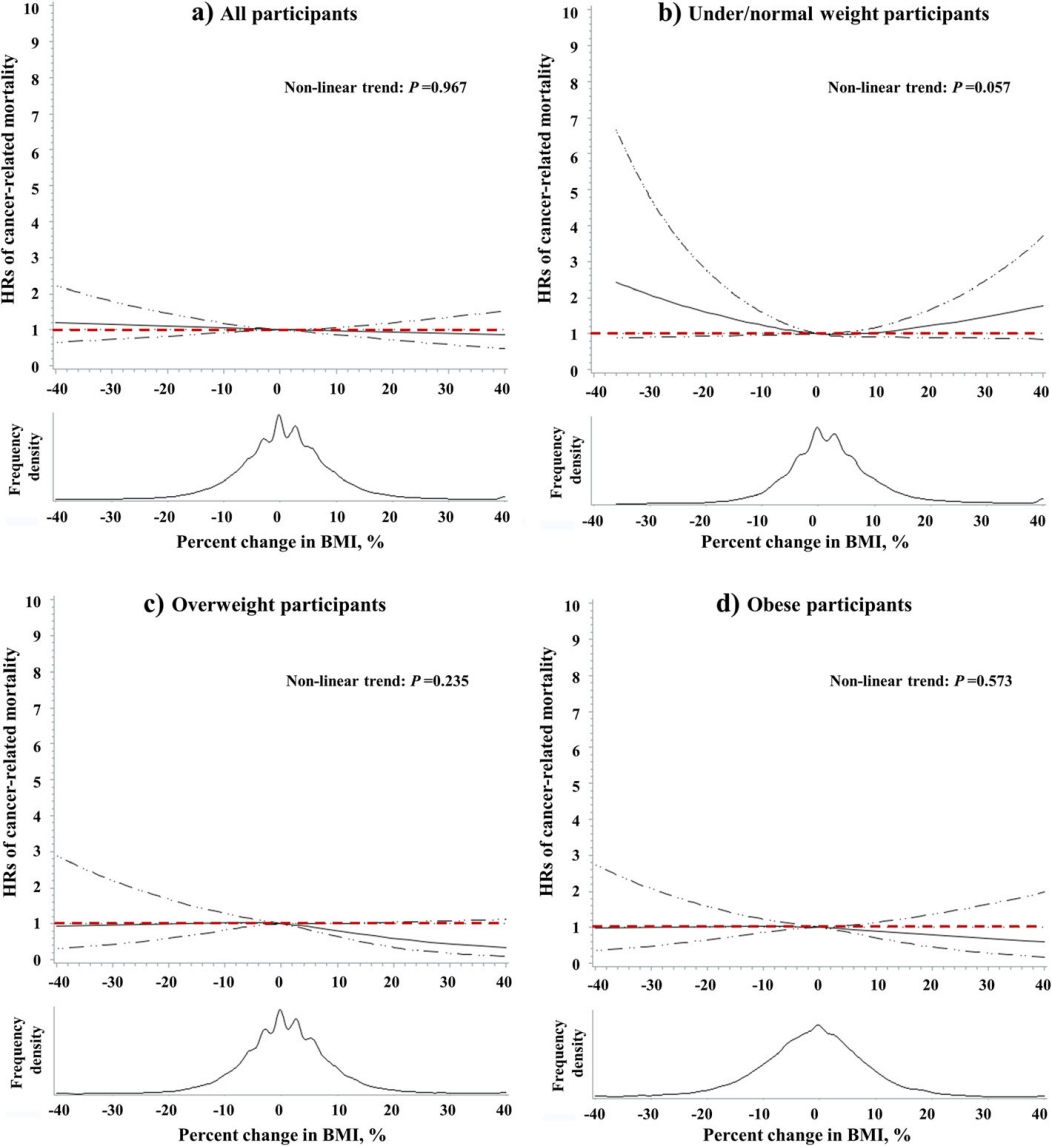
Restricted spline curves for the associations between percentage change in BMI and cancer-related mortality among overall (**a**), under/normal weight (**b**), overweight (**c**) and obese (**d**) participants. The solid curve represents the multivariate-adjusted HRs calculated by restricted cubic splines with 3 knots at the 25th, 50th, and 75th of the percentage change in BMI; the solid dashed lines represent corresponding 95% confidence interval. The reference value (HR = 1) was set at BMI percentage change = 0. HRs were estimated by cox proportional hazard model adjusted of sex, age, race, education level, family annual income, marital status, physical activity level, family history of cancer in their first-degree relatives, smoking status, screening arm, history of chronic diseases (i.e., hypertension, heart attack, stroke, emphysema, diabetes, arthritis, and osteoporosis), and BMI value at study entry (continuous)

Overall, the association between BMI change and the risk of cancer-related mortality was not statistically significant. We found significant interactions of sex (*p* for interaction = 0.016) and year of study enrolment (*p* for interaction = 0.003) with BMI change for the risk of cancer-related mortality. The trend analysis showed that a 5% decrease in BMI was associated with 14% (HR = 1.14, 95%CI: 1.02–1.27; *p* = 0.027) and 18% (HR = 1.18, 95%CI: 1.02–1.38; *p* = 0.042) increase in the risk of cancer-related mortality among men and those with > 10 years from study entry to 2006, respectively (Fig. 4). We did not find a significant nonlinear relationship between BMI change and the risk of cancer-related mortality among overall (*p* for nonlinear trend =0.967; Fig. 5a); among those who were under/normal weight (*p* for nonlinear trend = 0.057; Fig. 5b), overweight (*p* for nonlinear trend = 0.235; Fig. 5c), and obese (*p* for nonlinear trend = 0.573; Fig. 5d) participants, after adjustment of covariates.

### BMI change in relation to all-cause mortality

**Fig. 6:**
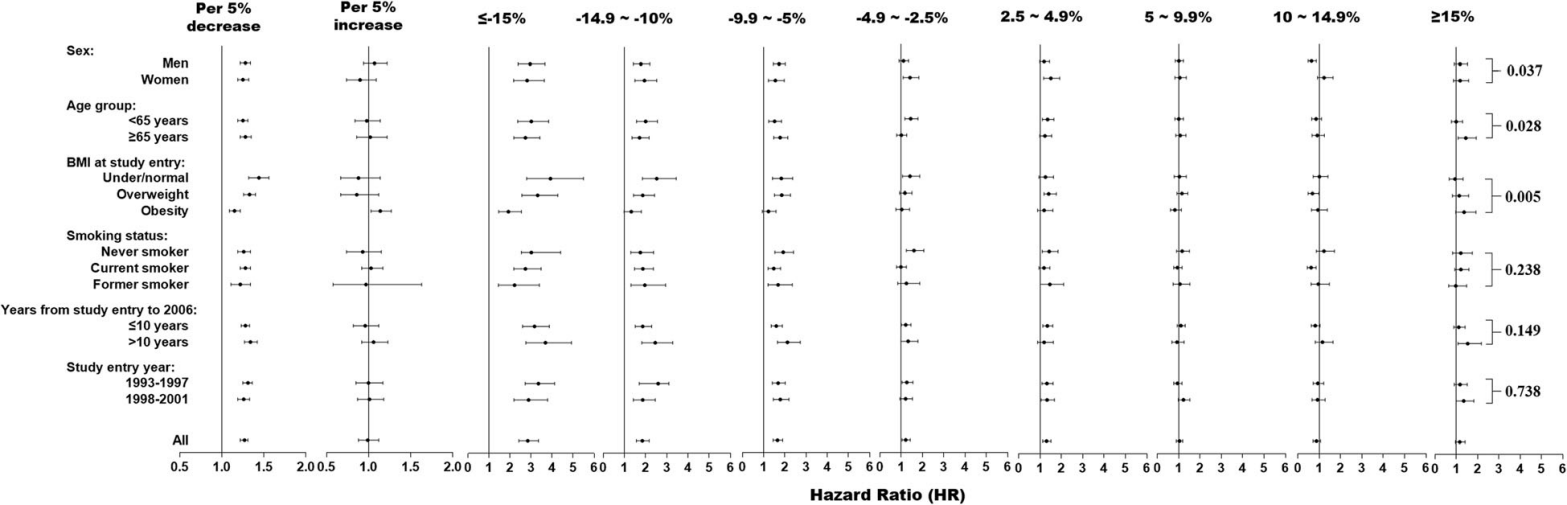
Associations between percentage change in BMI from study enrolment (1993–2001) to follow-up (2006) and the risk of all-cause mortality. The reference value (HR = 1) was set at percentage change between − 2.5 and 2.5%. HRs were estimated by cox proportional hazard model adjusted of sex, age, race, education level, family annual income, marital status, physical activity level, family history of cancer in their first-degree relatives, smoking status, screening arm, history of chronic diseases (i.e., hypertension, heart attack, stroke, emphysema, diabetes, arthritis, and osteoporosis), and BMI value at study entry (continuous)

As compared to participants whose BMI were stable, the HRs for participants who had 2.5–4.9%, 5.0–9.9%, 10.0–14.9%, and ≥ 15.0% decrease in BMI were 1.21 (95% CI: 1.03–1.42; *p* = 0.018), 1.65 (95% CI: 1.44–1.89; *p* < 0.001), 1.84 (95% CI: 1.56–2.17; *p* < 0.001), and 2.84 (95% CI: 2.42–3.35; *p* < 0.001) among overall participants, respectively. The subgroup analyses showed similar significant findings (Fig. 6).

Among participants who were overweight at study entry, those who became under/normal weight at follow-up had an 85% increased risk of all-cause mortality (HR = 1.85, 95% CI: 1.59–2.16, *p* < 0.001) as compared with those who were overweight both at study entry and follow-up. Among participants who were obese at study entry, those who became overweight or under/normal weight showed an increased risk of all-cause mortality (HR = 1.37, 95% CI: 1.13–1.67, *p* = 0.002 for overweight; HR = 2.59, 95% CI: 1.75–3.85, *p* < 0.001 for under/normal weight) when compared with those who were obese both at study entry and follow-up. (Table 2).

The trend analysis showed that a 5% decrease in BMI was associated with a 27% increase (HR = 1.27, 95%CI: 1.22–1.32; *p* for trend < 0.001) in the risk of all-cause mortality among overall participants. Subgroup analyses showed that the increased risks associated with 5% decrease in BMI ranged 15 to 44%. (Fig. 6).

**Fig. 7:**
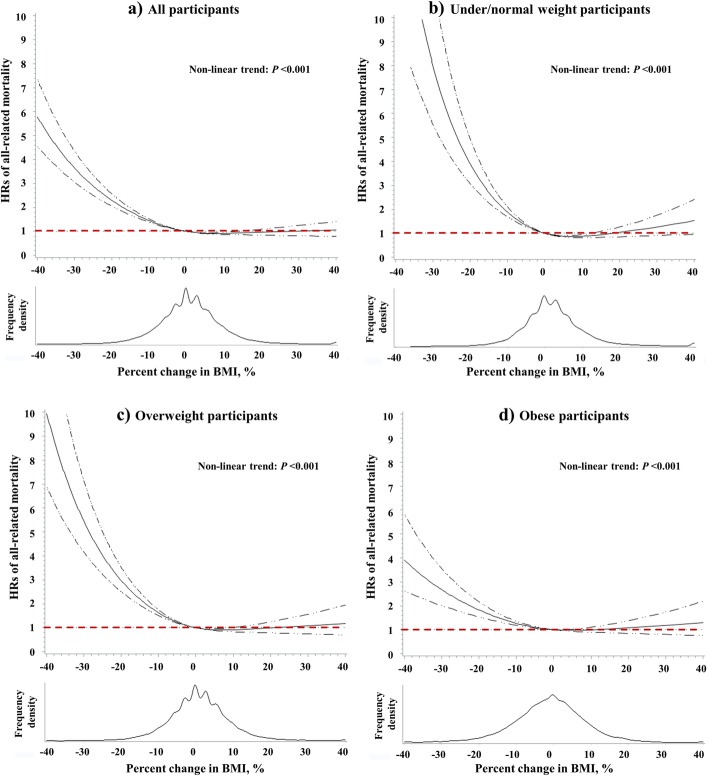
Restricted spline curves for the associations between percentage change in BMI and all-cause mortality among overall (**a**), under/normal weight (**b**), overweight (**c**) and obesity (**d**) participants. The solid curve represents the multivariate-adjusted HRs calculated by restricted cubic splines with 3 knots at the 25th, 50th, and 75th of the percentage change in BMI; the solid dashed lines represent corresponding 95% confidence interval. The reference value (HR = 1) was set at percentage BMI change = 0. HRs were estimated by cox proportional hazard model adjusted of sex, age, race, education level, family annual income, marital status, physical activity level, family history of cancer in their first relatives, smoking status, screening arm, history of chronic disease (i.e., hypertension, heart attack, stroke, emphysema, diabetes, arthritis, and osteoporosis), and BMI value at study entry (continuous)

A significant nonlinear relationship was observed between BMI change and all-cause mortality among overall (*p* for nonlinear trend < 0.001; Fig. 7a); among those who were under/normal weigh (*p* for nonlinear trend < 0.001; Fig. 7b), overweight (*p* for nonlinear trend < 0.001; Fig. 7c), and obese participants (*p* for nonlinear trend < 0.001; Fig. 7). The restricted cubic spline regression showed that the risk of all-cause mortality sharply increased with a decrease in BMI, but was not associated with an increase in BMI.

## Discussion

Using a large-scale data from the PLCO screening program of 81,388 midlife and elder individuals aged 55–74 years, we found that a decrease in BMI before cancer diagnosis was associated with an increased risk of all-cause mortality, but not for increase in BMI. Decrease in BMI was not significantly associated with the risk of CRC incidence and cancer-related mortality. In addition, the association between BMI changes and all-cause mortality indicated an L-shaped relationship, irrespective of the baseline BMI. Overall, a 5% decrease in BMI was found to be associated with a 15–44% increase in the risk of all-cause mortality.

The observed association between weight loss and the increased risk of mortality is consistent with findings from previous studies which focused on both midlife and old-aged adults [19, 27]. A meta-analysis containing 26 prospective studies reported that unintentional weight loss may be associated with 22–39% of weight loss-mortality risk [28]. It has been reported that the loss of lean mass may account for nearly a quarter of weight loss among 885 adults with impaired glucose regulation aged 60 to 90 years [29]. Considering that participants enrolled in this study were midlife to elderly individuals aged from 55 to 74 years, their loss of weight may intensify age-related lean mass loss, leading to physical function impairment [30]. Also, weight loss usually happens along with malnutrition, especially micronutrient deficiencies, and is accompanied by bone mineral density loss among the middle and the old-aged people [31]. Both mechanisms might account for the increased risk of mortality associated with weight loss. As compared to weight loss, weight gain was only associated with an increased risk of cancer-related or all-cause mortality among some subgroups; and in overall, weight gain was not significantly associated with all-cause mortality. Previous evidences from prospective studies indicated a reverse J-shaped association between weight change and the risks of both all-cause and cancer-related mortality [19, 28, 32, 33]. In a multiethnic 10-year prospective cohort study of 63,040 individuals aged 45–75 years, they found that increases in the risk of all-cause mortality were greater with weight loss than those with weight gain, indicating a reverse J-shaped association [33]. One reason for such inconsistency might be the lower sensitivity of weight gain to a short-term risk of mortality. As previous studies reported, weight gain could increase the likelihood of system inflammation, which could in turn lead to chronic diseases, such as cancer, cardiovascular disease, and diabetes mellitus [34]. Considering the long course of chronic diseases, the short-term risk of mortality might not increase. In other word, that means the long-term chronic disease and mortality would be largely decreased, if the weight gain or weight-gain related effects could be well managed during this short body reaction time, such as controlling weight, diet and healthy behaviors. Additionally, it is hinted that the avoirdupois monitoring among older population is a basic and critical tool for self-control and health management.

We did not find significant associations between weight change and the risk of CRC incidence or cancer-related mortality. The development of CRC is multifactorial, consisting of contributions from lifestyle habits and genetic factors. Body weight change might only partially reflect alteration of lifestyle habits, such as dietary intake and physical activity. Another possible explanation is the implementation of population-based screening program. Through several modalities (e.g., colonoscopy, fecal-based tests, and sigmoidoscopy), CRC is highly preventable if it is early diagnosed and treated [35, 36], leading to lower mortality in the general population. The third possible reason may be due to small sample size in some categories in our study. Although it is unclear which protective factors were associated with weight gain, it is widely reported that substantial degree of weight gain would lead to adipocyte hypertrophy, insulin resistance and obesity-related diseases, which could finally lead to higher mortality risk [37].

Considering the influence of baseline weight level, we calculated percentage change of BMI during the follow-up period. Also, after being stratified by BMI status at study entry, the obesity paradox for all-cause mortality was observed among participants who were normal/underweight, overweight, and obese. Increasing risk of all-cause mortality was found to be significantly higher in participants who were overweight/obesity at study entry and became under/normal weight at follow-up, and those who were obese at study entry and then became overweight at follow-up. In summary, those people who showed a decrease in weight have higher risk in all-cause mortality. It seems beneficial for midlife to elderly individuals to maintain a stable and slightly overweight BMI as they grow older. Considerable weight change during older life span, especially weight reduction, might not be recommended. As reported by Al Snih S et al, older adults with a BMI between 25 and 35 (typically overweight and even obese) had a lowest mortality [38].

Our study is based on a large-scale prospective cohort study involving subjects at their midlife to older ages. As a randomized trial, its design and data quality are robust. However, there are several limitations in our study. First, weight and height were self-reported both at recruitment and follow-up, and this may lead to misclassification bias. Second, we do not know whether participants went through an intentional weight-loss, although there might apply to only a minority of the population. Duration of obesity may be another important factor influencing our findings [20], and the effect of long duration of obesity on morbidity and mortality is definitely different from that of short-term duration. Third, as we excluded those with cancer diagnosis and those who were dead before follow-up (at 2006) because of the limitations of the study database, there might exist a healthy worker effect. Moreover, although we excluded the individuals who had history of cancer at study entry or were newly diagnosed with cancers before 2006, and also fully adjusted the potential confounding from personal history of chronic diseases, we could not completely rule out the confounding of chronic diseases related to BMI loss on death. Fourth, due to small number of incident CRC cases in some strata, the association between BMI change and the risk of CRC incidence should be interpreted with cautions. Fifth, BMI may be not a good indicator of adiposity in older individuals. Other body composition markers (e.g., waist circumference, waist-to-hip circumference) were not included; the combination of other markers and BMI would be helpful for further delineation of the effect of weight change on the risk of morbidity and mortality. Sixth, subjects who died in the period of observation might suffer from medical conditions that directly influence their BMI, such as cancer, stroke and diseases that could lead to sarcopenia. Finally, because this is a secondary analysis of the data from a randomized controlled trial, we could not absolutely exclude the “regression to the mean” effect.

## Conclusions

Our study comprehensively evaluated the associations between BMI change (both decrease and increase in BMI) before cancer diagnosis and the risks of CRC incidence, cancer-related mortality, and all-cause mortality in a large-scale midlife to elderly population. The findings suggest that decrease in weight among individuals, independent of chronic diseases, significantly increase the risk of all-cause mortality, but were not associated with the risk of CRC incidence and cancer-related mortality. Further studies are highly warranted to clarify the L-shape associations between weight change and the risk of mortality by considering more body composition markers at a long-time frame.

## Data Availability

The data that support the findings to this study are available from National Cancer Institute (NCI) but restrictions apply to the availability of these data, which were used under license for the current study, and so are not publicly available. Data are however available from the authors upon reasonable request and with permission of National Cancer Institute (NCI). No additional data are available.

## Abbreviations

95% CI: 95% Confidence interval
BMI: Body Mass Index
CRC: Colorectal Cancer
HRs: Hazard ratios
PLOC: Prostate, Lung, Colorectal, and Ovarian
SD: Standard deviations
